# Initiation Factor 3 is Dispensable For Mitochondrial Translation in Cultured Human Cells

**DOI:** 10.1038/s41598-020-64139-5

**Published:** 2020-04-28

**Authors:** Ivan V. Chicherin, Maria V. Baleva, Sergey A. Levitskii, Erdem B. Dashinimaev, Igor A. Krasheninnikov, Piotr Kamenski

**Affiliations:** 1 M.V. Lomonosov Moscow State University, Faculty of Biology, 119234 Moscow, Russia; 2 M.V. Lomonosov Moscow State University, Institute of Functional Genomics, 119234 Moscow, Russia; 3 Center for Genome Technologies, Pirogov Russian National Research Medical University, Moscow, Russia

**Keywords:** Molecular biology, Translation

## Abstract

The initiation of protein synthesis in bacteria is ruled by three canonical factors: IF1, IF2, and IF3. This system persists in human mitochondria; however, it functions in a rather different way due to specialization and adaptation to the organellar micro-environment. We focused on human mitochondrial IF3, which was earlier studied *in vitro*, but no knock-out cellular models have been published up to date. In this work, we generated human HeLa cell lines deficient in the MTIF3 gene and analyzed their mitochondrial function. Despite the lack of IF3mt in these cells, they preserved functional mitochondria capable of oxygen consumption and protein synthesis; however, the translation of ATP6 mRNA was selectively decreased which compromised the assembly of ATP synthase. Together with the analogous results obtained earlier for baker’s yeast mitochondrial IF3, our findings point to a functional divergence of mitochondrial initiation factors from their bacterial ancestors.

## Introduction

Mitochondria are double-membrane, essential organelles of eukaryotic cells responsible for aerobic respiration – a complex chain of redox reactions resulting in the production of ATP, the main cellular energetic currency, which fuels practically all biological processes. They are deeply integrated into the metabolic and signaling pathways of the cell, taking part in the citric acid cycle, heme and steroid synthesis, ROS and calcium ions signaling, apoptosis, etc. Mitochondrial dysfunctions are a common source of human disease^1^.

Mitochondria originated from α-proteobacteria billions of years ago, according to the endosymbiotic theory^2^. Their genomes lost the vast majority of genes that migrated into the nucleus in the course of evolution; therefore, these organelles are heavily dependent on the supply of proteins from cytoplasm through sophisticated import channels^3^. However, several protein-coding genes are retained in mammalian mitochondrial DNA, which suggests that the organelles preserved the functional machinery for their expression.

Translation in mitochondria follows the classical scheme with initiation, elongation, termination, and recycling steps^4^. Initiation of protein synthesis in bacterial cells is governed by three canonical factors: IF1, IF2, and IF3. These play an essential role in the recruitment of ribosomes on the mRNA, and the correct selection and positioning of the start codon and initiator tRNA^5^. The activities of these factors can still be recognized in mammalian mitochondria; however, now they have highly diverged from their ancestors both structurally and functionally^4^. Thus, IF1 and IF2 activities are combined in one polypeptide, IF2mt^6^. Mitochondrial IF3 (IF3mt) shows poor sequence homology to bacterial IF3, at the same time sharing its spatial organization: both bacterial and mitochondrial factors comprise two globular domains (N- and C-) connected with a helical linker^7^. Additionally, IF3mt has N- and C-terminal mitochondria-specific extensions. This factor specifically binds the 28S small mitochondrial ribosomal subunit and all its domains provide this interaction, contrary to prokaryotic IF3, where only the C-domain is involved, whereas the N-domain and the linker make no contacts with the subunit^8^. The overall position of IF3mt on the 28S subunit is consistent with its bacterial homolog: it localizes on the platform in the vicinity of the P-site^9^. IF3mt has two major activities *in vitro*: (1) binding the 28S subunit, and (2) dissociating 55S ribosomes^10^. Previous investigations demonstrated that they are organized rather differently in mitochondrial and prokaryotic systems. Mutagenesis studies showed that these activities are spatially separated in the IF3mt, which is not the case for IF3^11^.The structural elements are also exploited distinctly in these two factors. Thus, separate C-domain of IF3 can fulfill the role of the factor, whereas its N-domain only modulates its work^12^. In contrast, the activity of the separate C-domain of IF3mt is significantly reduced compared to the full-size protein; at the same time, the N-domain and the linker can bind the 28S subunit^10^. These data indicate that the mechanisms of action of IF3 and IF3mt are different despite their homology and structural similarity.

Despite the available data about the IF3mt protein, no studies describing the phenotype of the deletion of the MTIF3 gene in human cells have been published. In the Online GEene Essentiality database (OGEE, http://ogee.medgenius.info) we found the information about seven published large-scale CRISPR/Cas9 and RNAi screenings indicating that the deficiency of the MTIF3 gene should not compromise the survivability and the growth of the human cultured cells. On the other hand, there is a report of the International Mouse Phenotyping Consortium (IMPC) that MTIF3 gene disruption generated in mice had a deleterious phenotype: heterozygous mutants demonstrated hypoactivity and homozygous mutation resulted in the preweaning lethality, and complete penetrance (https://www.mousephenotype.org/data/genes/MGI:1923616). No targeted research characterizing the phenotypic effects of MTIF3 knock-out on the molecular level in human cells has yet been conducted. In the current work, we generated MTIF3 knock-out human cell lines and analyzed their mitochondrial function.

## Results

### A knock-out of the MTIF3 gene impairs the proliferation and viability of human cultured cells

**Figure 1:**
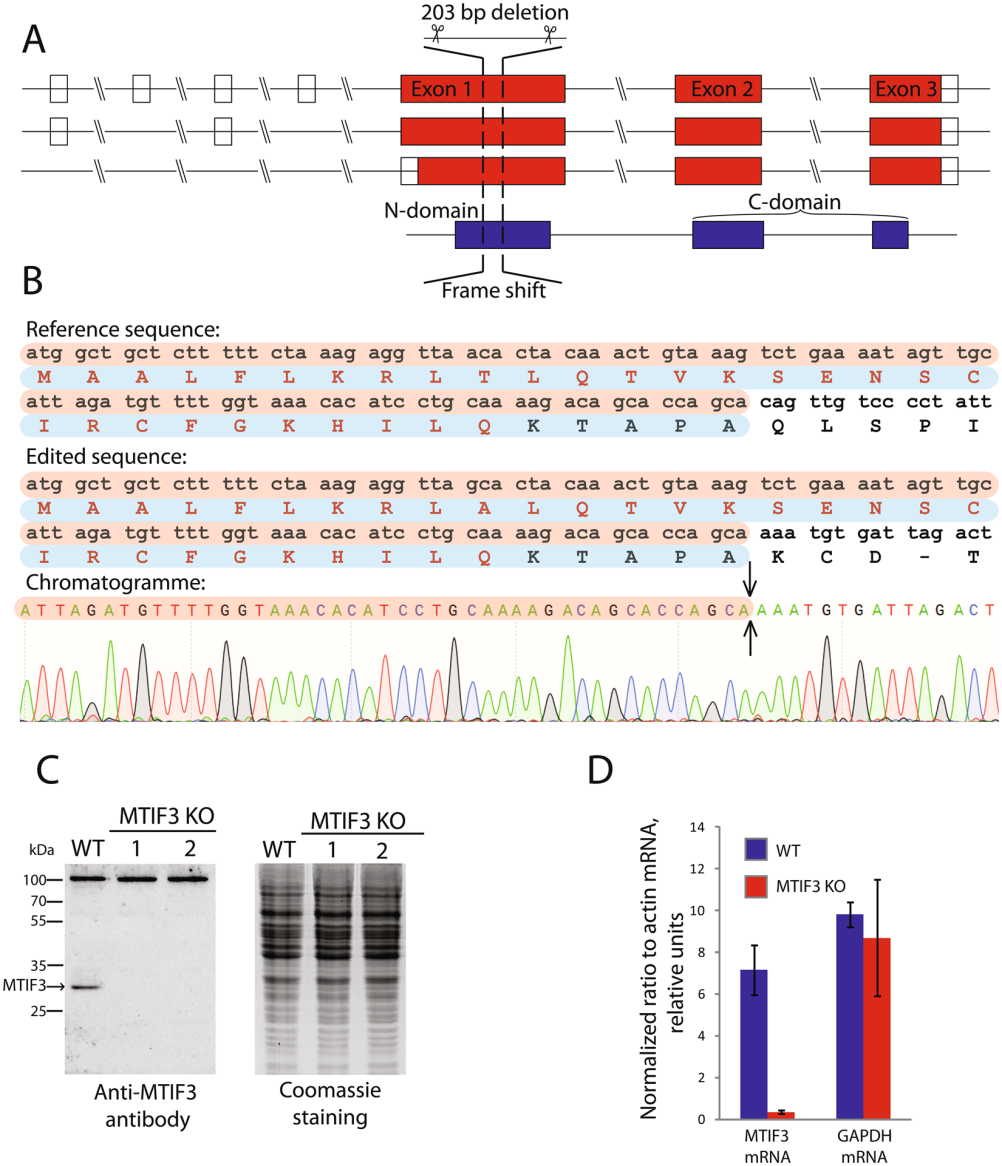
Disruption of MTIF3 gene in HeLa cells. (**A**) The structure of the MTIF3 gene is represented according to the Ensembl genome browser. Three alternatively spliced mRNAs (red) and one protein (blue) are shown. Transcripts are drawn as boxes (exons) and lines connecting the boxes (introns). Filled boxes represent coding sequence and unfilled boxes (or portions of boxes) represent UnTranslated Regions (UTR). For coding transcripts (red), protein motifs and domains are shown in blue. Two crossing parallel lines indicate the long genome regions of the introns which were not shown to keep the scale of the scheme. (**B**) The results of the sequencing of the DNA of the mutant cells. The coincident DNA sequences are in brown boxes, coincident protein sequences are in blue boxes. Protein sequences typed with red letters are the mitochondrial transit peptide (MTS). The sequences start from the first ATG codon and first methionine respectively. The region of the mutant gene where the deletion takes place is indicated by an arrow. Only partial sequences demonstrating the frame-shift are shown. (**C**) The results of the IF3mt immunodetection by Western blot (left panel). WT: wild type cells; MTIF3 KO: mutant cells with a disrupted MTIF3 gene; 1 and 2 are separate clones. The IF3mt band is marked by an arrow. The positions of the protein molecular weight markers together with their weights in kDa are depicted on the left. As a loading control, the gel with the same samples loaded was stained by Coomassie R250 (right panel). (**D**) RT-qPCR analysis of MTIF3 gene expression. WT: wild type cells; MTIF3 KO: mutant cells with disrupted MTIF3 gene. GAPDH gene expression serves as an internal control. The data were calculated following the ((Ct algorithm and normalized on the signal of the β-actin mRNA primer pair. The error bars were built based on three independent experiments.

According to the data found in the genome browser Ensembl (https://www.ensembl.org), the expression of the human MTIF3 gene gives rise to several mRNAs produced by alternative splicing (Fig. 1A). All of them have three major protein-coding exons: exon 1, encoding 31 amino acid long mitochondrial targeting sequence (MTS) and the N-domain, as well as exons 2 and 3 encoding the C-domain. We applied the CRISPR/Cas9 genome editing technique to knock-out the MTIF3 gene. We designed two pairs of guide RNAs intended to cut the DNA locus of exon 1 and to generate the deletion of 203 bp in the coding region, which should produce a frame-shift soon after the start of translation, leaving only the MTS of IF3mt fused with several amino acids from the mature part of the protein (Fig. 1B). The sequences were inserted in gRNA expression vectors, which were used for the transfection of HeLa cells as described in Materials and Methods. From about 30 clones analyzed, two homozygous clones with both MTIF3 alleles disrupted were selected. To confirm the MTIF3 gene disruption, the DNA of the selected clones was analyzed by PCR (Supplementary Figure 1) and Sanger sequencing (Fig. 1B). The absence of the IFmt protein was verified by Western blot (Fig. 1C), and the absence of the corresponding mRNA was confirmed by RT-qPCR (Fig. 1D). All these tests showed that the both alleles of the MTIF3 gene were disrupted and the IF3mt protein was absent in two mutant clones.

**Figure 2:**
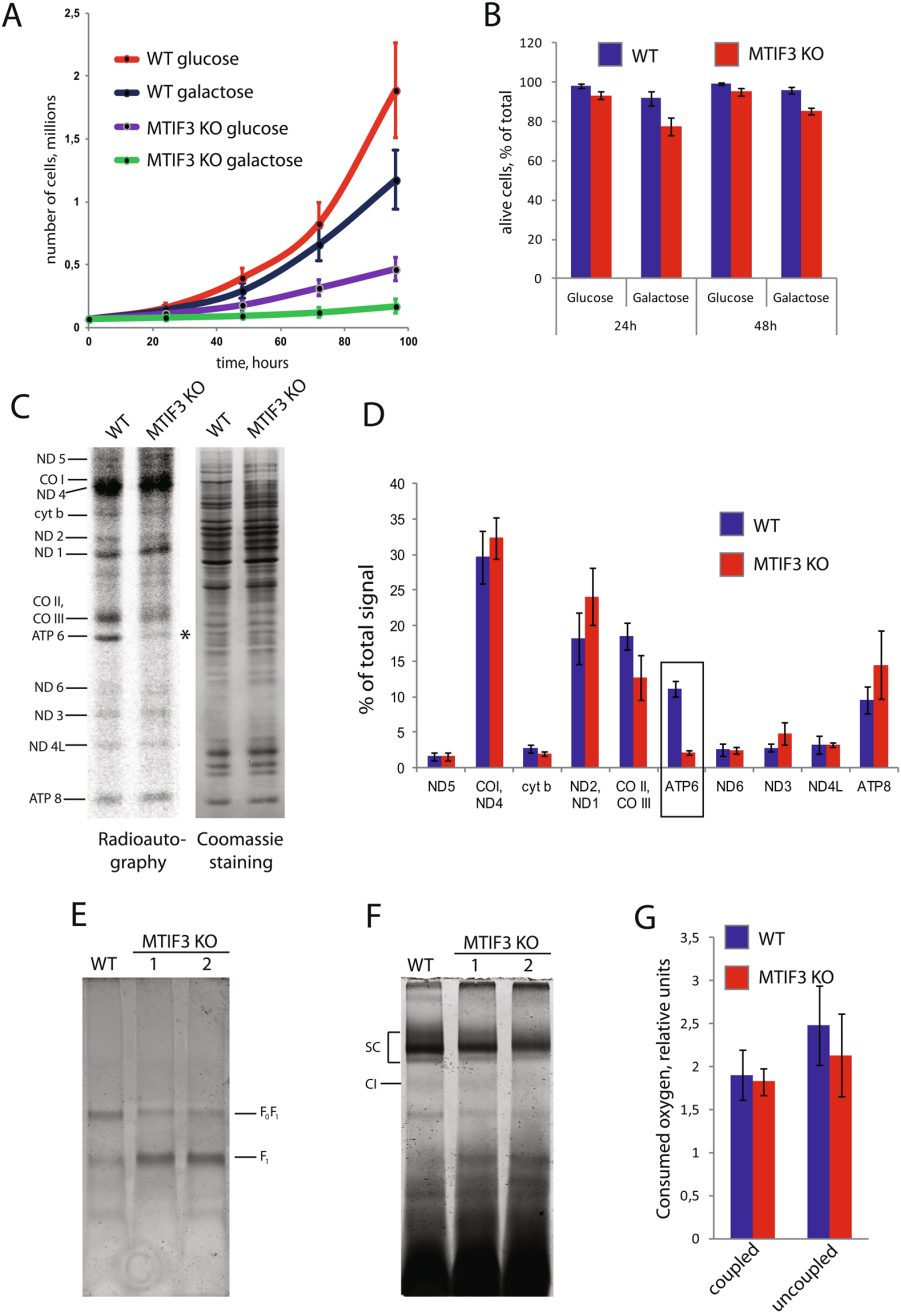
Analysis of the mitochondrial function of MTIF3 knock-out cells. The designations are the same for all panels (WT: wild type cells; MTIF3 KO: mutant cells with disrupted MTIF3 gene; 1 and 2 are separate clones). (**A**) Growth curves of WT and MTIF3 KO cells in glucose- or galactose-containing media. The error bars were built based on three independent experiments. (**B**) Analysis of the cell viability on glucose- or galactose-containing media in the indicated periods of time. The error bars were built based on three independent experiments. (**C**) The radioautograph of labeled mitochondrial translation products separated by electrophoresis in 15–20% gradient PAAG (left panel). Cell lines used for the experiment are depicted on the top. Individual mitochondrial proteins are marked on the left. The asterisk marks the band of ATP6. As loading control, the gel was stained with Coomassie R250 (right panel). The experiment was done three times; the typical picture is shown. (**D**) Calculations of the relative quantities of mitochondrial translation products shown in (**C**). The frame marks the reduction of ATP6 amount as a result of MTIF3 gene disruption. The error bars were built based on three independent experiments. (**E**) The staining of complex V activity. Cell lines used for the experiment are depicted on the top. The positions of complete F0F1 enzyme and free F1 subunit are indicated. (**F**) The staining of complex I activity. Cell lines used for the experiment are depicted on the top. SC: supercomplexes, CI: free complex I. The weak bands below free complex I are the residual signals of the complex V staining. (**G**) Oxygen consumption in the wild type HeLa and MTIF3 mutant cells. Quantification of coupled and uncoupled respiration is presented (indicated on the bottom). The error bars were built based on four independent experiments.

To access possible phenotypic consequences of MTIF3 gene knock-out, we probed the proliferation and viability of mutant HeLa cells. For checking the proliferation, we monitored growth on two types of DMEM media, either glucose- or galactose-containing. The cultivation in DMEM-galactose forces the cells to rely almost entirely on mitochondria for ATP production while the presence of glucose in the medium makes mitochondrial function less important for the cells. We demonstrated pronounced growth defects of MTIF3 knock-out cells in the standard DMEM medium with either glucose or galactose (Fig. 2A). It is important to note that the growth on galactose was remarkably slower than that on glucose. Also, the number of living cells monitored by trypan blue staining was lower in the population of MTIF3 knock-out HeLa in the galactose-containing medium (Fig. 2B). Taken together, a reduced proliferative ability and increased mortality of MTIF3 mutant cells in galactose media relative to the growth of the wild-type cell line suggest mitochondrial dysfunction.

### Knock-out of the MTIF3 gene selectively decreases translation of the ATP6 mRNA in mitochondria of human cultured cells

It is well-known that IF3 plays a central role in the ribosomal cycle and the corresponding gene is essential in prokaryotes^13^. On the other hand, the deletion of its homolog in yeast *S. cerevisiae* mitochondria, *AIM23*, did not block mitochondrial translation, but brought significant “imbalance” in protein synthesis^14^. Having human cell lines with MTIF3 deficiency in our hands, it was tempting to explore what happens with the human mitochondrial translation system upon the deletion of the IF3 homolog. A straightforward strategy is generally used to answer this kind of question^15^. The cells are incubated for a short time in the presence of the antibiotic cycloheximide which specifically blocks cytosolic translation, and then ^35^S-radioactively labeled methionine is added in the culture medium. The label is included selectively in mitochondrial translation products in these conditions, and since there are only 13 of them, they can be resolved by SDS-PAGE (Fig. 2C).

The overall efficiency of radioactive label incorporation in mitochondrial translation products of MTIF3 knock-out cells was close to the wild type cells (Fig. 2C), so, in this test, human mitochondrial translation behaved similarly to the yeast system rather than the prokaryotic one. The calculations of the relative intensities of the signals corresponding to each band demonstrated that the translation of ATP6 mRNA (encoding subunit 6 of ATP synthase F_0_) is selectively decreased in MTIF3 knock-out cells (Fig. 2D). Thus, the protein synthesis in human mitochondria in the absence of IF3mt became “imbalanced”; however, in a different way than in the yeast system, where the deletion of *AIM23* increased the synthesis of Atp6 and Atp9 products, and decreased CO I and CO II^14^.

### Knock-out of the MTIF3 gene compromises the assembly of F0F1 ATP synthase in the mitochondria of human cultured cells

We decided to investigate further if the decrease in ATP6 synthesis caused by MTIF3 deletion would affect the assembly and activity of ATP synthase (Complex V) in human mitochondria. For this, we isolated mitochondria from wild type HeLa cells and two clones bearing the MTIF3 deletion (1 and 2). We solubilized the organelles with digitonin, which extracts protein complexes in mild conditions, and separated them by blue native PAGE. The in-gel activity of complex V was assayed as described in Materials and Methods. Both mutant cell lines demonstrated the same results, showing a decrease of the F_0_F_1_ enzyme and an excess of the free F_1_ subunit (Fig. 2E). A similar phenotypic effect of ATP6 deficiency in human cells, when the assembly of complex V was impaired and the free F_1_ subunit accumulated, was described previously^16^. Based on our data, we build a model which implies that the lack of IF3mt drops the synthesis of ATP6, which, in turn, results in fewer quantities of the F_0_ subunit, and therefore impairs the assembly of F_0_F_1_ ATP synthase.

Respiratory chain complexes can assemble in higher-order structures with strictly defined stoichiometry called supercomplexes^17^. The exact role of these entities has been debated; however, there is no doubt that they are important for mitochondrial functions. Intriguingly, recent investigations demonstrated that the deletion of the MTIF3 homolog in yeast, *AIM23*, completely abolished the assembly of the supercomplexes in mitochondria^18^. We decided to explore whether or not the same thing happens in human cells. To visualize the supercomplexes, we stained the blue native gel described above by a NADH-NBT redox reaction, which stains free complex I and its superassemblies (I + III_2_, I + III_2_ + IV and other of uncertain composition). Our data show that MTIF3 deletion did not hinder their assembly (Fig. 2F).

### Knock-out of the MTIF3 gene does not affect the oxygen consumption of human cultured cells

Quite often, dysfunctions in mitochondria cause the respiratory defects. This is not surprising, as respiration relies heavily on mitochondria. This was observed for *AIM23*, the homolog of IF3 in yeast mitochondria: the strain bearing *null* deletion had a pronounced lag phase growing in media with non-fermentable carbon sources^14^. To assess the respiratory function of MTIF3 knock-out cells, we measured their oxygen consumption in two modes, namely coupled and uncoupled. Coupled respiration shows oxygen consumption under the physiological conditions of the work of the electron transfer chain, whereas in the uncoupled mode, the cells are treated with protonophore, making the mitochondrial membrane permeable for H^+^ ions. The latter reflects the full capacity of complex IV, which works independently from the proton gradient and ATP synthesis in these conditions. Our results show that a lack of IF3mt does not affect the oxygen consumption in either mode (Fig. 2G), which is rather surprising regarding the opposite effect of the absence of Aim23p in yeast.

### The MTIF3 deletion phenotype is rescued by the extrachromosomal expression of the MTIF3 gene

**Figure 3:**
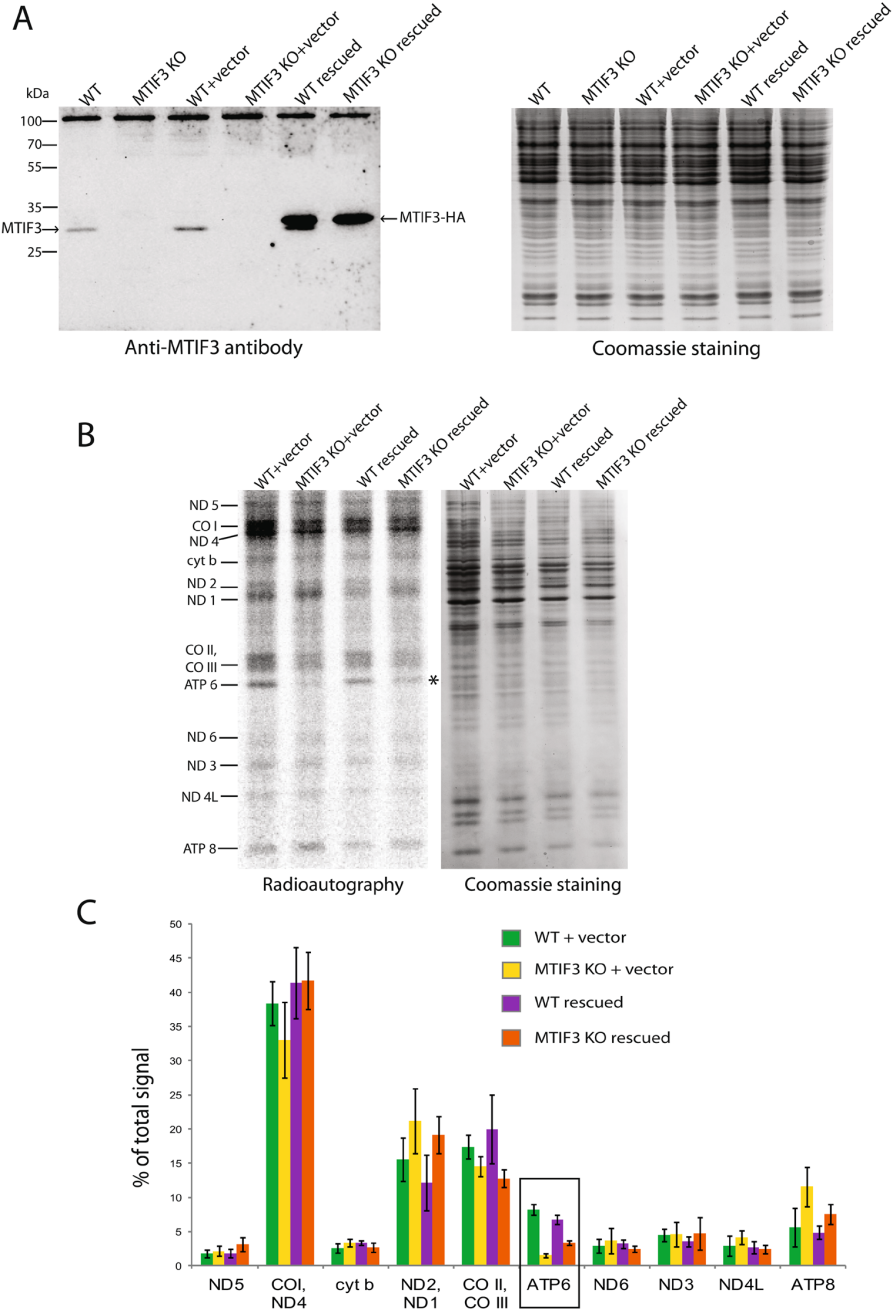
Analysis of the mitochondrial function of MTIF3 knock-out cells rescued by the MTIF3-coding plasmid. The designations are the same for all panels (WT + vector: wild type cells transfected with the empty vector; MTIF3 KO + vector: mutant cells with disrupted MTIF3 gene transfected with the empty vector; WT rescued: wild type cells transfected with the MTIF3-coding plasmid; MTIF3 KO rescued: mutant cells with disrupted MTIF3 gene transfected with the MTIF3-coding plasmid). (**A**) The results of the IF3mt immunodetection by Western blot (left panel). Cell lines used for the experiment are depicted on the top. The bands of the native IF3mt and the fusion protein IF3mt-HA are marked with the arrows. The positions of the protein molecular weight markers together with their weights in kDa are depicted on the left. As a loading control, the gel with the same samples loaded was stained by Coomassie R250 (right panel). (**B**) The radioautograph of labeled mitochondrial translation products separated in 15–20% gradient PAAG (left panel). Cell lines used for the experiment are depicted on the top. Individual mitochondrial proteins are marked on the left. The asterisk marks the band of ATP6. As loading control, the gel was stained with Coomassie R250 (right panel). (**C**) Calculations of the relative quantities of mitochondrial translation products shown in (**B**). The frame marks the partial restoration of the ATP6 amount in mutant cells as a result of the MTIF3 synthesis from the plasmid. The error bars were built based on three independent experiments.

To show the specificity of the described effect we did a rescue experiment. For this, we inserted the human MTIF3 gene fused to the sequence coding for the HA-tag in the mammalian expression vector pcDNA5/FRT/TO. The construct was transfected in MTIF3 knock-out cells. As controls, we transfected the wild type and MTIF3 knock-out cells with the same vector, or an empty vector. The expression of the gene was verified by Western blot (Fig. 3A). From the immunoblotting results, it can be concluded that the MTIF3 gene is overexpressed from the plasmid, which may be linked to the strong CMV promoter presence within the vector.

We then analyzed the mitochondrial translation profiles in the above-mentioned cell lines (the radioautograph is presented on Fig. 3B). The signals of individual mitochondrial proteins were calculated in Image J software and normalized on the overall mitochondrial translation (Fig. 3C). We conclude that the expression of MTIF3 in the knock-out cells partially restores the level of ATP6 synthesis. It is worth mentioning that this restoration does not reach the values of the wild type cells, probably due to the cytotoxic effect of transfection manifested by the lower cell recovery in the samples of MTIF3 knock-out cells. The amounts of several other mitochondrial proteins had also changed in response to transfection with the rescue plasmid; however, partial restoration of ATP6 is the only statistically significant event, as it appears on the diagrams in Fig. 3C. We also were not able to detect any meaningful change in *de novo* synthesized mitochondrial proteins after transfection of our cell lines with the empty vector. This means that the increase of the ATP6 synthesis rate is indeed the consequence of IF3mt reappearance in the MTIF3 knock-out cells. Finally, we detected no significant effects in wild type cells transfected with the rescue plasmid. Obviously, MTIF3 overexpression itself does not alter mitochondrial translation; thus, the observed rescue effect should be attributed to the re-occurrence of MTIF3 in the cells rather than to its overexpression.

## Discussion

In this study, we assessed the influence of MTIF3 gene deletion on mitochondrial translation in cultured human cells. The mammalian IF3mt protein was studied *in vitro* in recent decades; however, the knock-out experiments in cultured human cells dedicated to this gene have not yet been conducted. Using the CRISPR-Cas9 technology, we generated the MTIF3 knock-out lines of HeLa cells and analyzed their mitochondrial translation.

The MTIF3 gene turned out to be non-essential for the survivability of human cultured cells even when both alleles were deleted, but this deletion compromised growth in the standard complete DMEM media with glucose or galactose. At the same time, the deletion of the MTIF3 homolog in yeast *S. cerevisiae*, *AIM23*, was also non-lethal and the growth of the mutant strain was slower on non-fermentable media^14^. On the other hand, bacterial IF3 is essential because its deficiency blocks the progression of the ribosomal cycle^13^. Conversely to the cell model which we described in this report, the disruption of the MTIF3 gene in mice carried out by the International Mouse Phenotyping Consortium was lethal for the animal in the homozygous state. This was recently confirmed in an article by Rudler *et al*.^19^ published during the revision of our work. This means that the effects of MTIF3 knockout manifest differently in cultured cells and in developing embryos. The difference may come from the role which IF3mt plays during some stages of development, the process far more complex than the proliferation of cultured cells. At the same time, the embryos with both MTIF3 alleles deleted pass through several early stages of development reaching day 8.5^19^, meaning that cell proliferation persists even in MTIF3 knockout cells.

Rudler and colleagues also generated a heart- and skeletal muscle-specific *Mtif3* knock-out mouse line and demonstrated the misbalanced mitochondrial translation in the heart and muscle cells of these animals. The effect was not the same as we showed: the authors saw an overall increase of the translation rate, as well as an increase or decrease of several individual mitochondrial protein synthesis rates. We think that this is not exclusive to our results. First, the molecular effect of an absence of MTIF3 may be differently manifested in cultured human cells and animal models. Second, Rudler and colleagues performed mitochondrial translation analysis *in organello*, using isolated mouse heart mitochondria, while we worked with intact human cells from another tissue. Finally, the ATP6 band is poorly visible on mitochondrial translation profiles by Rudler *et al*., making its quantitative evaluation almost impossible. Overall, we believe that our results are generally in line with the results of Rudler and colleagues: in both works, it has been demonstrated that (1) mitochondrial translation in the absence of MTIF3 is possible but misbalanced, and (2) MTIF3 knock-out has phenotypic consequences.

The lack of IF3mt did not change drastically the overall efficiency and the pattern of protein synthesis in the mitochondria of cultured human cells, with the only exception being down-regulation of ATP6. The expression of MTIF3 from the plasmid partially rescued this deletion phenotype. The specific effect of the deletion on the ATP6 product can be explained by the unusual feature of the corresponding ORF. There are only two cases of overlapping ORFs in human mitochondrial DNA^20^. One of them is the ATP6 ORF which has a 46 nucleotide overlap of its 5′-end with the 3′-end of the preceding ATP8 ORF. Another case is the ND4 ORF having its first 7 nucleotide overlapped with the 3′-end of the preceding ND4L ORF. Correspondingly, two bicistronic mRNAs, namely ATP8/ATP6 and ND4L/ND4, are presented in human mitochondria among typical monocistronic mRNAs. Little is known about the translation initiation of these messengers. Given the difference in the sizes of the overlapping regions, one can suggest that the ATP6 and ND4 ORFs may exploit different translation initiation pathways, and IF3mt may play significant role in the synthesis of ATP6 but not ND4. The role of MTIF3 in such putative “internal initiation” should be studied in future.

Yeast and mammalian mitochondria lacking IF3mt are capable of protein synthesis; this means that mitochondrial IF3s functionally diverge from their bacterial ancestors. On the other hand, the exact molecular effects of IF3mt absence are different in yeast, mice and cultured human cells^14,19^. This may be explained by the evolutional diversification of mitochondrial translation systems. Yeast mitochondrial mRNAs are characterized by the presence of long untranslated regions (UTRs) containing dozens or even hundreds of nucleotides at both the 5′- and 3′-ends; they also lack polyA sequences^20^. On the contrary, mammalian mitochondrial mRNAs almost lack the 5′-UTRs (3 nt is the maximum length) and are polyadenylated. The regulation of mitochondrial translation in yeast is carried out by the unique system of translational activators, a set of proteins each of which governs the translation of only one mRNA^20^. This system is unique for budding yeast and is not found in mammals.

MTIF3-deficient cell lines demonstrated decreased amounts of functional F_0_F_1_ ATP synthase and increased amounts of free F_1_ subunit. Thus, the decrease of ATP6 expression impaired the assembly of the ATP synthase. An analogous effect was previously described for the mutation in ATP6^16^. However, the electron transfer chain supercomplexes were unaffected in the mutant cells, conversely to what was observed in yeast in an analogous situation, when all supercomplexes disappeared^18^. The lack of IF3mt did not alter the oxygen consumption of human cultured cells, meaning that the work of the electron transfer chain and complex IV were close to the conditions of the wild type cells.

**Figure 4:**
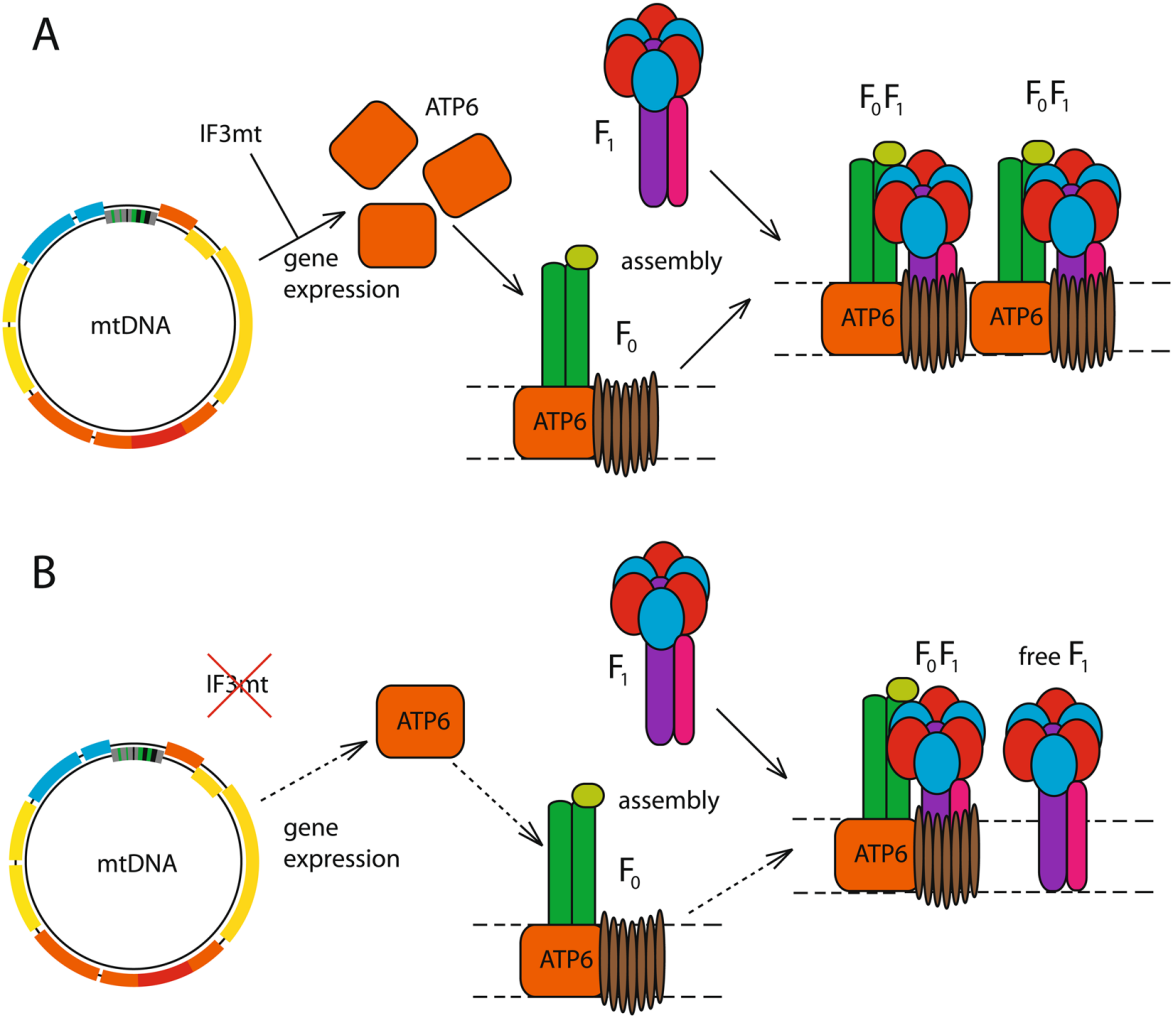
The proposed model explaining the effect of MTIF3 deletion in human cells. (**A**) The situation in the wild type cells when MTIF3 is expressed. (**B**) The situation in MTIF3-deficient cells. See Discussion for the details.

In summary, our data show that human mitochondrial translation initiation is closer to the yeast one rather than to the prokaryotic ancestors. However, translation systems function differently in yeast and human mitochondria. Despite the deficiency in IF3mt, mutant human cells possess functional mitochondria with properly assembled supercomplexes and are capable of respiration and protein synthesis. Based on our findings, we propose a model explaining the observed phenotype of MTIF3 deletion in human cultured cells (Fig. 4). Thus, the lack of IF3mt decreases the expression of ATP6 in mitochondria, which in turn results in lower amounts of the F_0_ subunit of ATP synthase, limiting the assembly of the F_0_F_1_ complete enzyme. It not evident at the moment why the deficiency of IF3 in both yeast and human mitochondria selectively affects the expression of only certain genes, but this definitely means that the factor works differently than in prokaryotic protein synthesis.

## Materials and Methods

### Plasmids

The plasmid pX330-U6-Chimeric_BB-CBh-hSpCas9 was a gift from Feng Zhang (Addgene plasmid # 42230; http://n2t.net/addgene:42230; RRID: Addgene_42230;^21^). The plasmid pCas9-IRES-EGFP was obtained by subcloning the Cas9 ORF from the plasmid pX330-U6-Chimeric_BB-CBh-hSpCas9 to the vector pIRES2-EGFP (Clontech, USA) in the MCS. This part of the work was carried out in the Evrogen company (Moscow, Russia). The plasmid pU6-gRNA^22^ was kindly provided by Dr. Skryabin (Munster University, Germany). The sequence of the MTIF3 gene with the HA-tag at the 3-terminus was amplified from the cDNA of HeLa cells with primers 5′-ctgtggatccatggctgctctttttctaaag-3′ and 5′-ctgtctcgagttaagcgtaatctggaacatcgtatgggtactgatgcagaacatttg-3′ and cloned into a pcDNA5/FRT/TO vector (Invitrogen, USA) at BamHI and XhoI sites.

### Cell culture

HeLa cells (ATCC CRM-CCL-2) were maintained in Dulbecco′s Modified Eagle′s Medium (DMEM, Gibco, Thermo Scientific, USA) supplemented with 10% fetal bovine serum (Corning, USA), 4 mM glutamine, 1 mM sodium pyruvate, and 100 U/ml penicillin-streptomycin (Thermo Scientific, USA) at 37 °C in an atmosphere of 5% CO_2_. For the detachment, cells were washed with Versene solution (Thermo Scientific, USA) and treated with 0.05% Trypsin-EDTA (Thermo Scientific, USA). Cells were transfected either by electroporation on Gene Pulser Xcell (Bio-Rad, USA) or by lipofection with Lipofectamine 3000 (Thermo Scientific, USA).

### Analysis of proliferative activity and viability of the cells

The series of approximately 70000 cells were placed in the culture dishes (three for each line and condition to acquire the statistics) and cultivated in two different media. One was DMEM high glucose (4.5 g/L) (Gibco, Thermo Scientific, USA) supplemented with 10% FBS (Corning, USA), 4 mM glutamine, 1 mM sodium pyruvate and 100 U/ml penicillin-streptomycin (Thermo Scientific, USA); the other was DMEM with no glucose medium (Thermo Scientific, USA) supplemented with 10% FBS (Corning, USA), 4 mM glutamine, 1 mM sodium pyruvate and 100 U/ml penicillin-streptomycin (Thermo Scientific, USA) and 4.5 g/L of galactose (filter sterilized). For the proliferative activity, the amount of cells was calculated every 24 hours on an automated TC-20 cell counter (Bio-Rad, USA) over the course of 4 days. For the viability, 24- and 48-hour time points were used. The content of living cells was calculated on the same device by staining with 0.4% Trypan Blue solution (Bio-Rad, USA).

### CRISPR/Cas9-mediated disruption of MTIF3 gene

The variable parts of guide RNA sequences were designed in the Benchling online platform (https://benchling.com). The corresponding DNA sequences were cloned into a pU6gRNA vector. The sequences of these variable parts are 5′-GCAATAGGGGACAACTGTGC-3′ (gRNA1) and 5′-GAAGTCTAATCACATTTGCT-3′ (gRNA2). HeLa cells were transfected with the mixture of these two gRNA vectors, as well as with a pCas9-IRES-EGFP plasmid bearing the Cas9 nuclease and an EGFP fluorescent marker. For transfection, Lipofectamine 3000 (Thermo Scientific, USA) was used. In 48 hours after transfection, cells were selected on a FACSAria SORP cell sorter (BD Biosciences, USA). Individual clones were isolated with the standard method of ultimate dilutions. Their genomic DNA was extracted with phenol-chloroform and analyzed by PCR. The DNA locus bearing the intended deletion was amplified by PCR with primers 5′-GACTTCCTGTGTGTGGATC-3′ and 5′-CCTCTTGTCACAGGCACCT-3′ and cloned into a pTZ57R/T vector using a InsTAclonePCR Cloning Kit (Thermo Scientific, USA). Plasmid clones were isolated using Plasmid Miniprep (Evrogen, Russia) and Sanger sequenced (Evrogen, Russia).

### Western blot

The cells were harvested, washed and resuspended in 200 μl of 1x PBS. The suspension was sonicated on ice at 10% of maximal amplitude for 10 seconds, three times, with 3-minute chilling intervals on a Branson Sonifier 250 (Branson, USA). Protein concentration in the sample was measured by Bradford assay. Equal amounts of protein (30 μg per lane) were loaded on a 12% Laemmli SDS-PAAG. Upon electrophoresis completion, the proteins were transferred from the gel to an Amersham Protran nitrocellulose membrane (Sigma Aldrich, USA). The membrane was stained with primary and secondary antibodies as described in^23^. The primary antibodies to MTIF3 were purchased from Proteintech (14219-1-AP); the secondary antibodies were ECL Anti Rabbit IgG HRP linked the whole antibody from donkey (GE Healthcare). The chemiluminescent signals were registered in ChemiDoc Imager (Bio-Rad).

### RT-qPCR

RNA was isolated from the tested cell lines with TRIZOL (Invitrogen, Thermo Scientific, USA). cDNA synthesis was done with M-MLV RT kit (Evrogen, Russia). Quantitative PCR was done with qPCRmix-HS SYBR (Evrogen, Russia) using a CFX96 Touch Real-Time PCR Detection System (Bio-Rad, USA). Calculations were done in CFX Manager Software (Bio-Rad, USA). We used the following primers: b-actin mRNA (5′-CACCATTGGCAATGAGCGGTTC-3′ and 5′-AGGTCTTTGCGGATGTCCACGT-3′), MTIF3 mRNA (5′-GCACCAGCACAGTTGTCC-3′ and 5′-CCCAAATCATTGCCCTTCTC-3′) and GAPDH mRNA (5′-GTCTCCTCTGACTTCAACAGCG-3′ and 5′-ACCACCCTGTTGCTGTAGCCAA-3′).

### Mitochondrial translation assay

*In vivo* ^35^S-labelling of mitochondrial translation products was done as described in^24^ with modifications. The cells were cultured in DMEM in 6 cm dishes until they reached approximately 70% confluence. The medium was changed for RPMI without methionine (Thermo Scientific) supplemented with 10% dialyzed FBS. The cytoplasmic translation was inhibited by treatment with 0.2 mg/mL cycloheximide for 5 min at 37 °C. Immediately after, 20 µCi of ^35^S-methionine (Perkin Elmer, USA) were added in the medium for 30 min at 37 °C. The reactions were stopped by the addition of cold methionine (20 mM) and puromycine (1 µg/ml). Next, the cells were collected, washed, resuspended in PBS buffer and briefly sonicated in for 10 s at 10% of maximum amplitude on a Branson Sonifier 250 (Branson, USA). The samples with equal amounts of protein (~75 µg of total protein per lane) were separated in gradient 15–20% SDS-PAAG. Upon electrophoresis completion, the gel was stained with Coomassie R-250 and dried. The signals were scanned in a Storm 865 Imager (GE Healthcare, USA). Calculations were done in ImageJ software^25^.

### In-gel activities of complexes I and V

We isolated mitochondria from the cells as described in^26^. Approximately 5 ×10^7^ cells were collected by centrifugation (400 g, 4 min), resuspended in 1.1 ml of an ice-cold RSB Hypo buffer (10 mM NaCl, 1.5 mM MgCl_2_, 10 mM Tris-HCl, pH 7.5) and left on ice for 5 minutes to swell. The cells were broken with 8 strokes of a Dounce glass homogenizer, and 0.8 ml of 2.5 x MS buffer (525 mM mannitol, 175 mM sucrose, 12.5 mM Tris-HCl, pH 7.5, 2.5 mM EDTA, pH 7.5) were added immediately after homogenization. The final volume was brought to 5 ml with 1 x MS buffer (210 mM mannitol, 70 mM sucrose, 5 mM Tris-HCl, pH 7.5, 1 mM EDTA, pH 7.5). The suspension was centrifuged at 1300 g for 5 minutes three times to remove nuclei and unbroken cells. Mitochondria were pelleted at 12000 g for 15 minutes and washed once with 1 x MS buffer. Mitochondrial proteins were solubilized with digitonin (6 mg per 1 mg of protein) and separated by Blue Native PAGE as described in^27^. Complex V activity was stained in-gel as described in^28^, with modifications. Briefly, the gel was washed with distilled water several times and incubated in 50 mM glycine for 1 h. Then it was stained in a medium containing 35 mM Tris-HCl pH 7.8, 270 mM glycine, 14 mM MgSO_4_, 5 mM ATP, and 0.2% (w/v) Pb(NO_3_)_2_ overnight with gentle shaking at room temperature. Complex I activity was stained in-gel as described in^28^, with modifications. Briefly, the gel was washed with distilled water for 1 minute at room temperature and then stained in the solution containing 0.1 M Tris-HCl pH 7.4, 1 mg/ml nitro blue tetrazolium (NBT), and 0.14 mM NADH overnight with gentle shaking at room temperature.

### Measurement of oxygen consumption

The test was done using a Clark oxygen electrode as described in^28^. The cells (approximately 5 ×10^6^) were collected, resuspended in 300 μL of DMEM and placed in a chamber of Oxytherm+ device (Hansatech, UK) at 37 °C with mixing. Coupled respiration was registered for 3 min 30 s. Next, the cells were treated with 2 µL of 10 mg/ml digitonin (Calbiochem) for 2.5 minutes, and then 2 µL of 1 mM carbonyl cyanide m-chlorophenylhydrazone (CCCP, Sigma, USA) was added. The uncoupled respiration was registered for 2 minutes. The reactions were stopped with 700 μM of KCN. Oxygen consumption was calculated in Oxytrace + software (Hansatech, UK) and normalized to the quantity of the cells calculated in a TC20 cell counter (Bio-Rad). Final values were represented in Units: 1U is 1 μM of consumed oxygen per 1 million cells per minute.

## Supplementary information

Supplementary information.
