# Supplementary material for: Initiation Factor 3 is Dispensable For Mitochondrial Translation in Cultured Human Cells
Source: Sci Rep. 2020 Apr 28;10:7110. doi: 10.1038/s41598-020-64139-5 (PMC7188818; doi:10.1038/s41598-020-64139-5)

SUPPLEMENTARY MATERIALS FOR

INITIATION FACTOR 3 IS DISPENSABLE FOR MITOCHONDRIAL TRANSLATION IN CULTURED HUMAN CELLS

Ivan V. Chicherin[1,2]*, Maria V. Baleva[1], Sergey A. Levitskii[1], Erdem B. Dashinimaev[3], Igor A. Krasheninnikov[1] and Piotr Kamenski[1]*

[1] M.V. Lomonosov Moscow State University, Faculty of Biology, 119234 Moscow, Russia.

[2] M.V. Lomonosov Moscow State University, Institute of Functional Genomics, 119234 Moscow, Russia.

[3] Center for Genome Technologies, Pirogov Russian National Research Medical University.

* Corresponding author e-mails: i.v.chicherin@gmail.com (Ivan V. Chicherin), peter@protein.bio.msu.ru (Piotr Kamenski).

Supplementary Figure 1. PCR amplification of the characteristic clones DNA after disruption of MTIF3 gene using CRISPR/Cas9 technology. PCR was performed with two primers flanking the desired deletion region (see Materials and Methods for their sequences). Heterozygous clone (one of about 15 we obtained) gives rise to two PCR products: 714 bp (corresponds to wild type DNA; marked with the asterisk) and 511 bp (corresponds to DNA with deletion; marked with the double asterisk). Homozygous clones 1 and 2 that had been further used in this work give rise to one PCR product of 511 bp. Correspondingly, wild type (WT) clone gives rise to one PCR product of 714 bp. Characteristic sized of the molecular weight fragments in base pairs are indicated on the left.

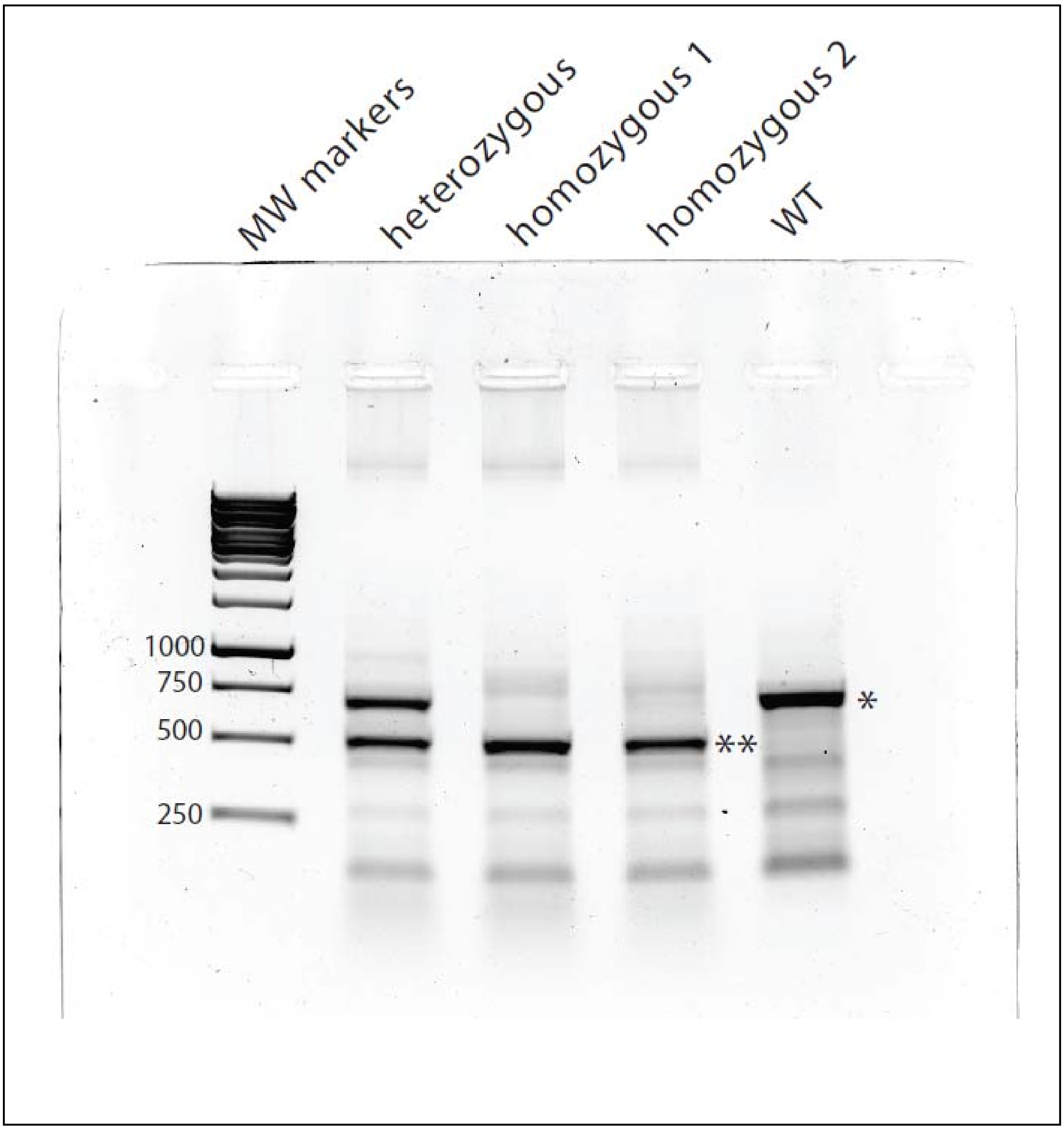

Supplementary Figure 2. Original image of the Figure 1C (Western Blot) in the article. The region of the original image used in the main figure is denoted with the red box.

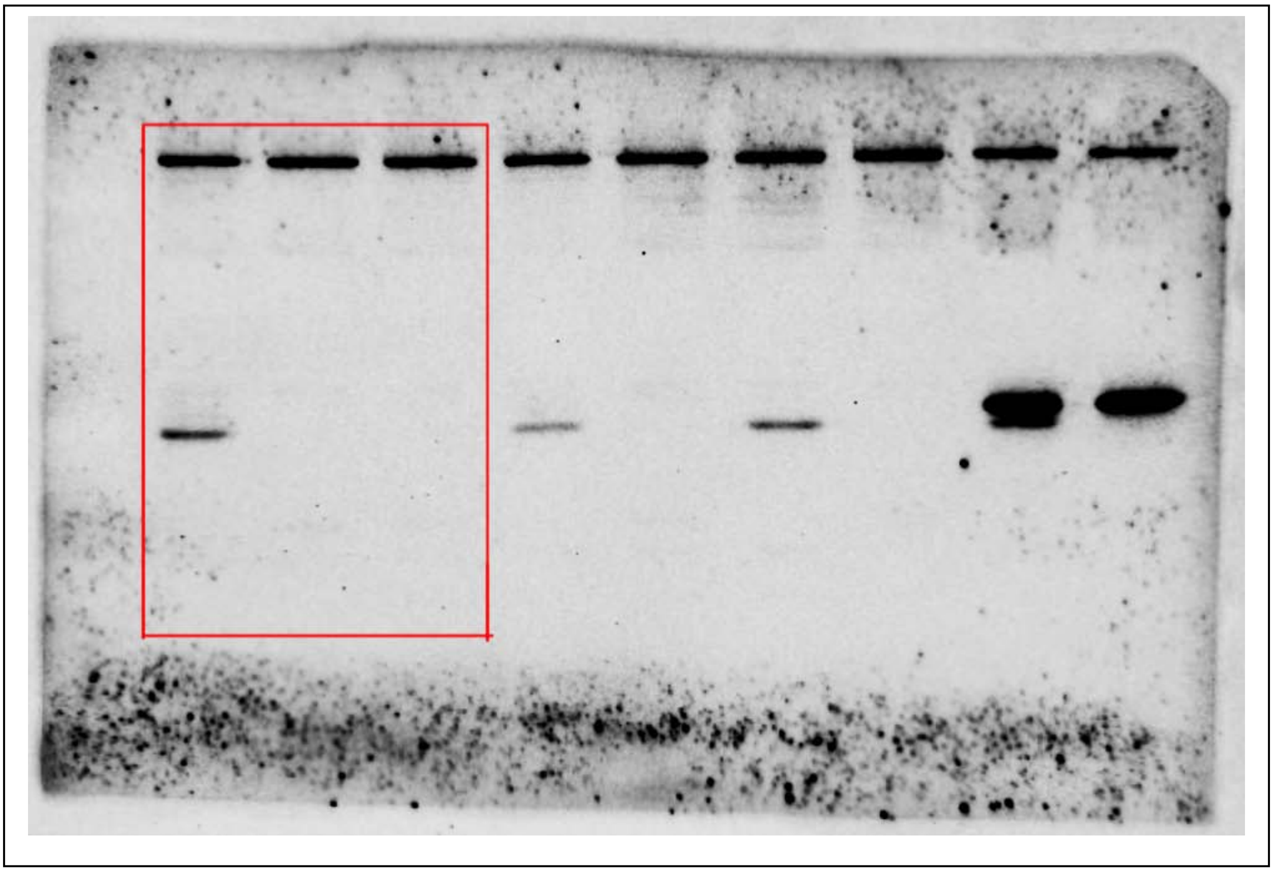

Supplementary Figure 3. Original image of the Figure 1C (Coomassie staining) in the article. The region of the original image used in the main figure is denoted with the red box.

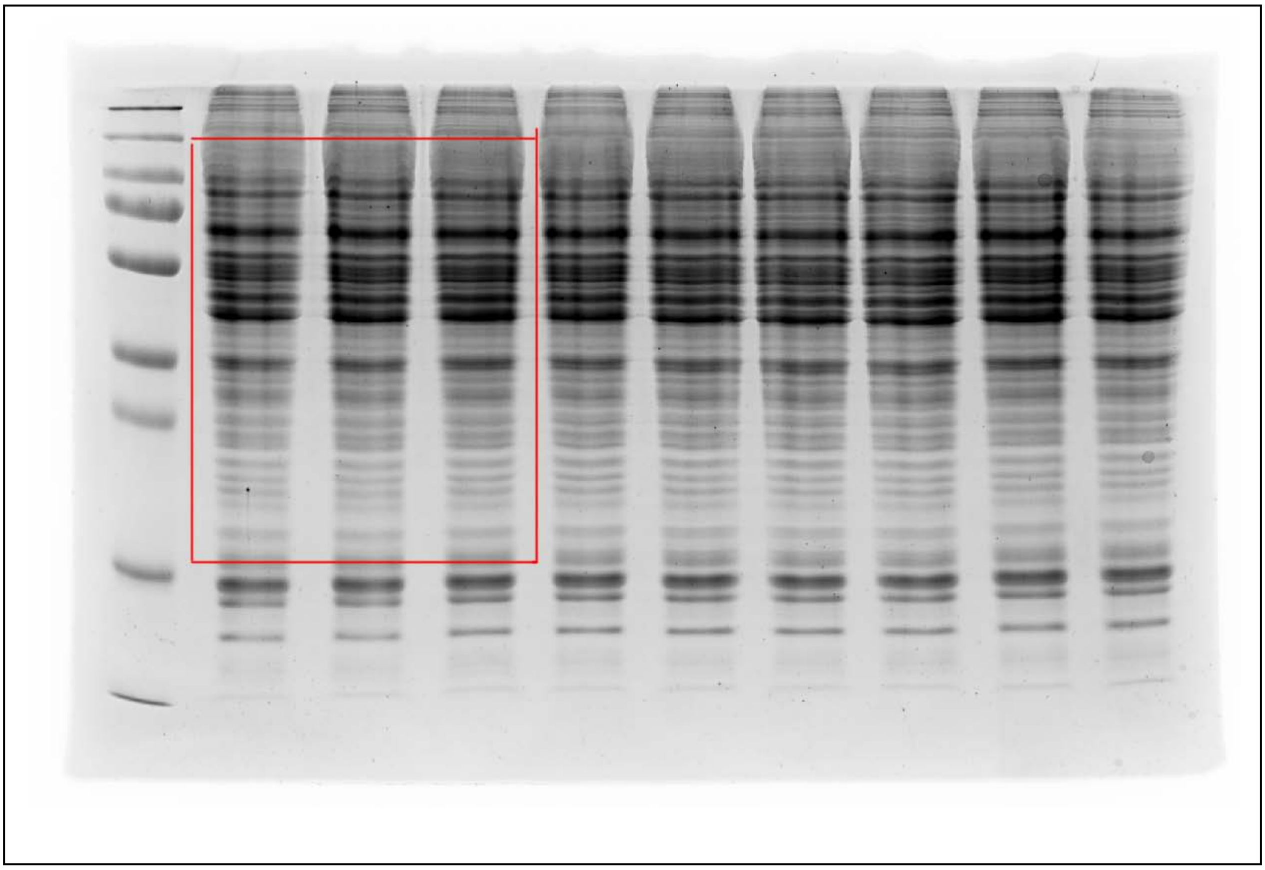

Supplementary Figure 4. Original image of the Figure 2C (radioautography) in the article. The region of the original image used in the main figure is denoted with the red box.

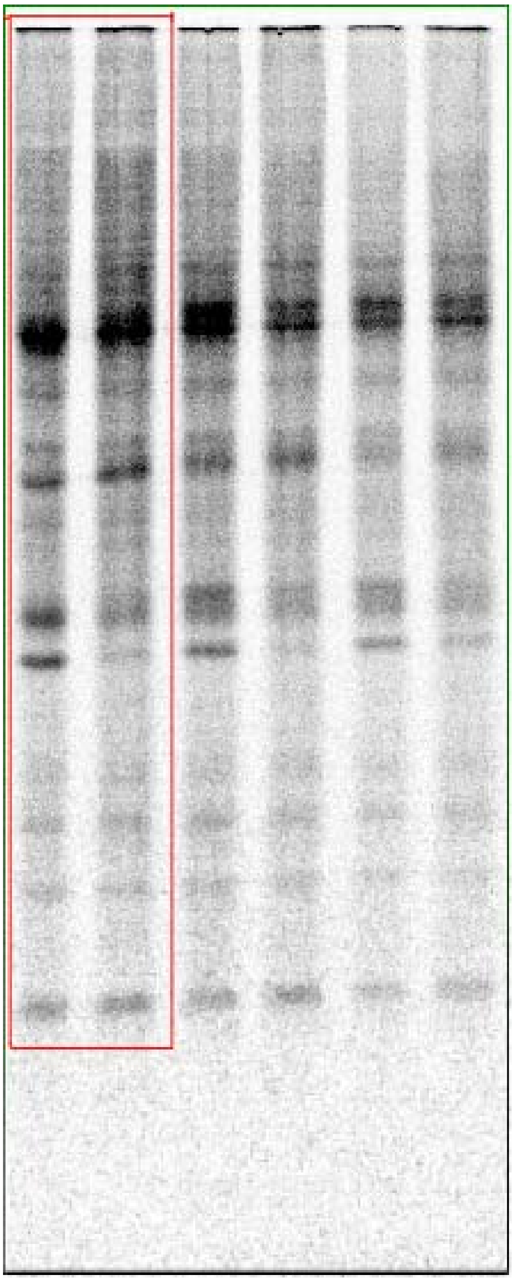

Supplementary Figure 5. Original image of the Figure 2C (Coomassie staining) in the article. The region of the original image used in the main figure is denoted with the red box.

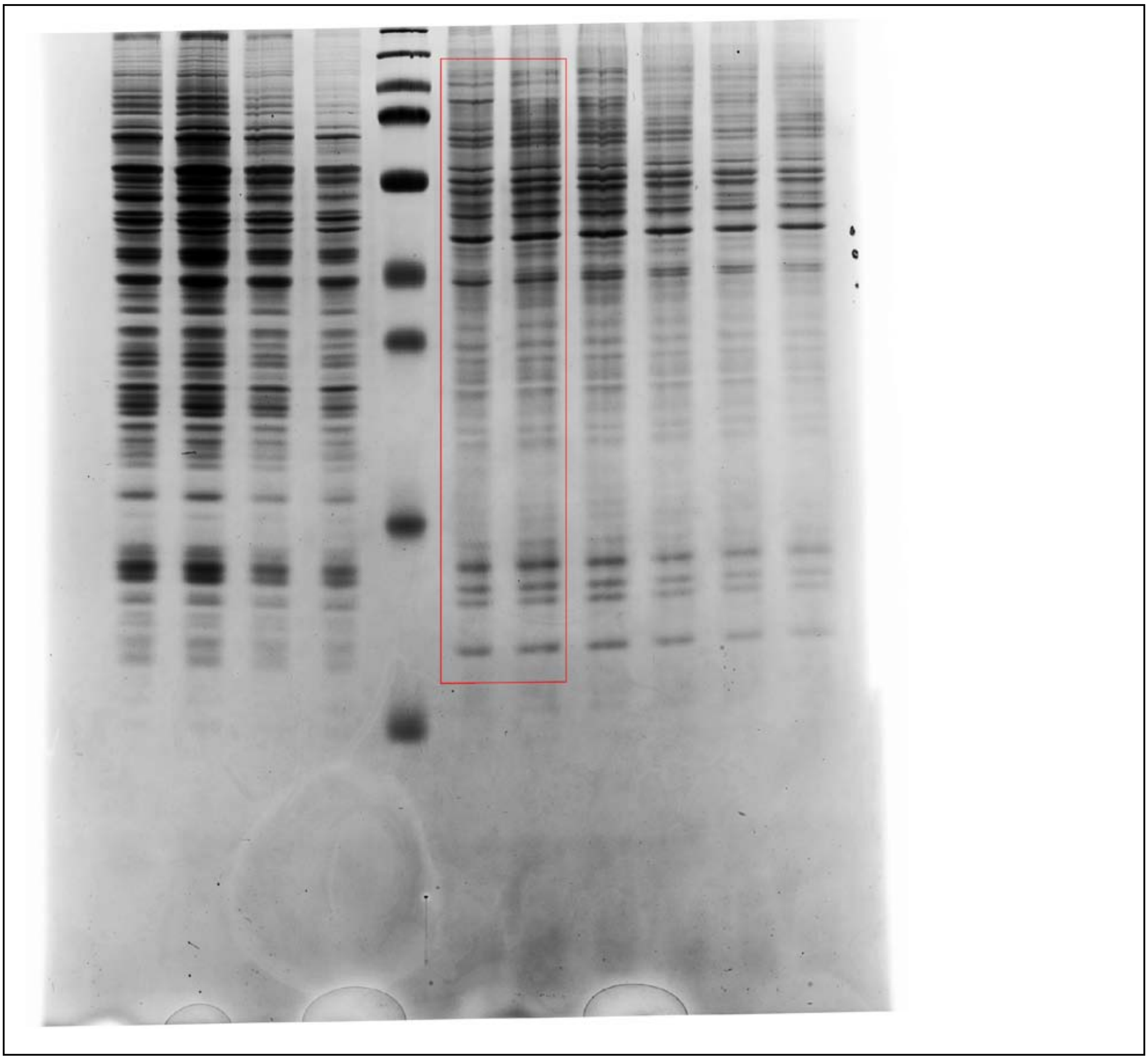

Supplementary Figure 6. Original image of the Figure 2E in the article. The region of the original image used in the main figure is denoted with the red box.

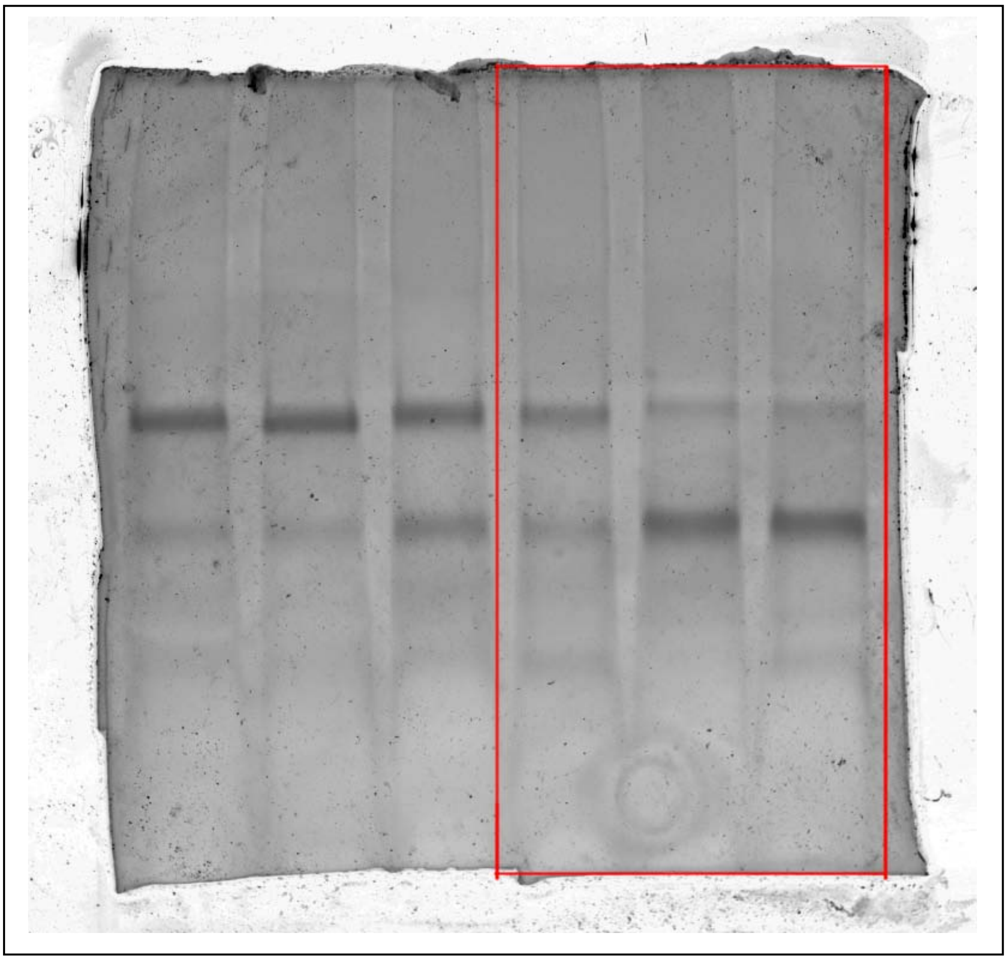

Supplementary Figure 7. Original image of the Figure 2F in the article. The region of the original image used in the main figure is denoted with the red box.

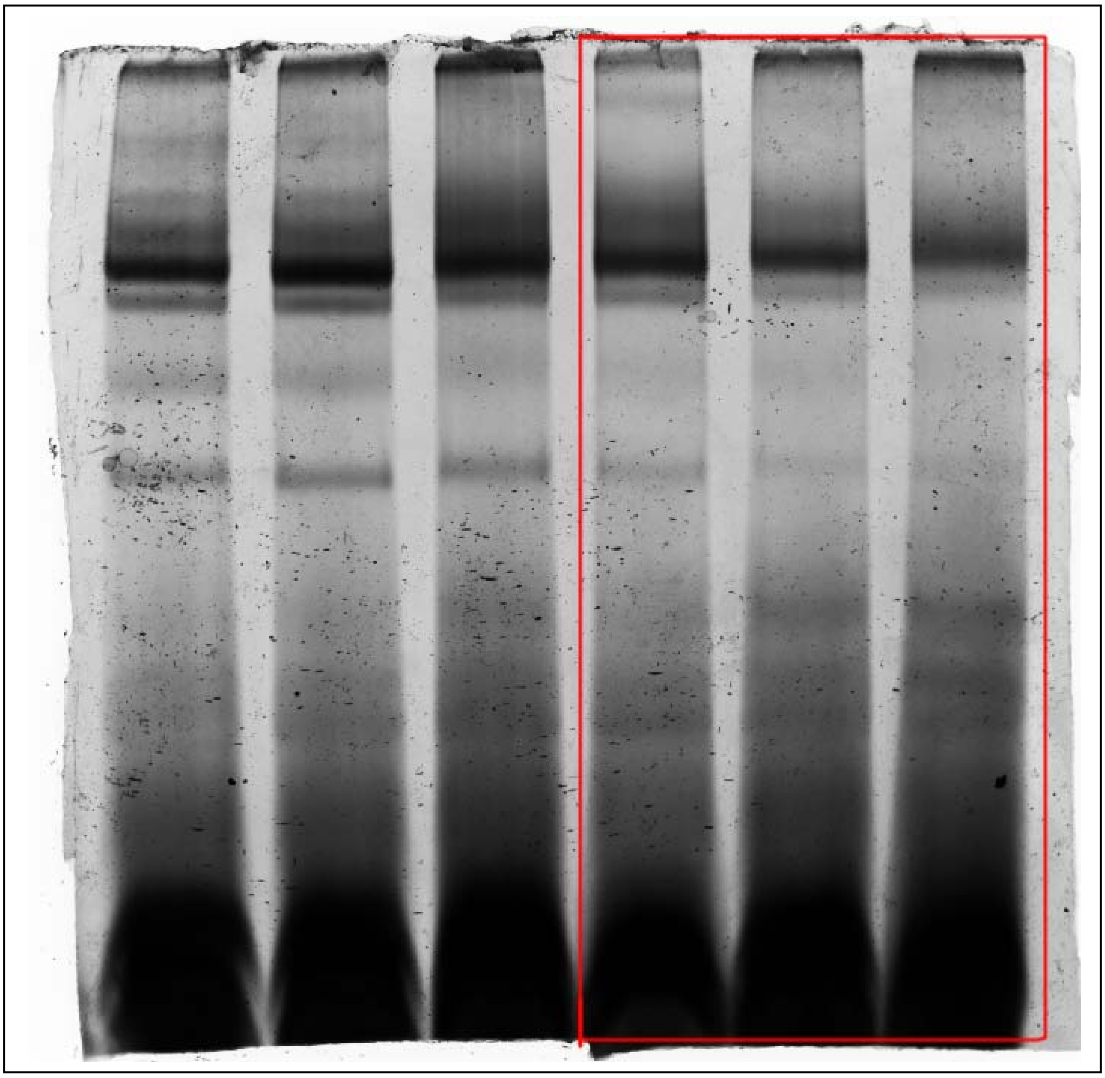

Supplementary Figure 8. Original image of the Figure 3A (Western Blot) in the article. The region of the original image used in the main figure is denoted with the red box.

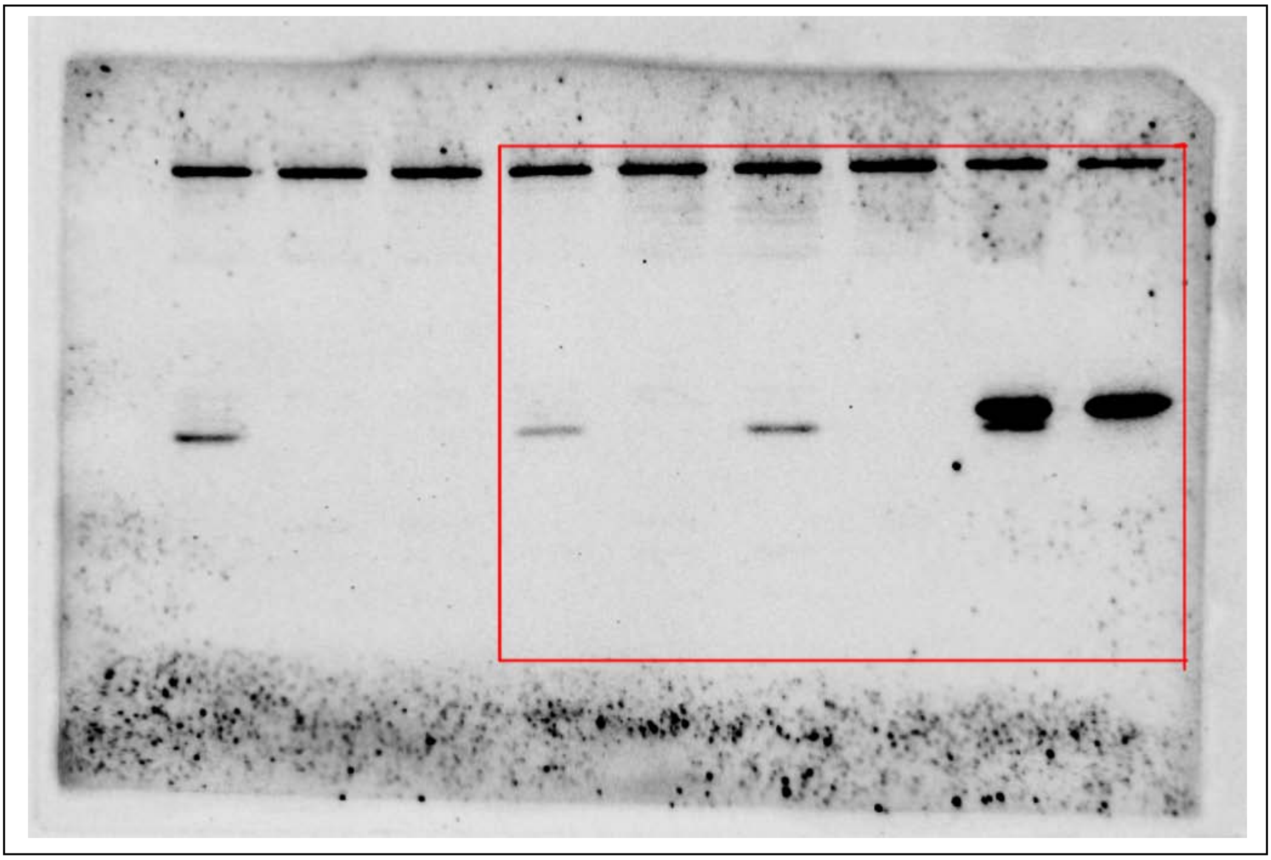

Supplementary Figure 9. Original image of the Figure 3A (Coomassie staining) in the article. The region of the original image used in the main figure is denoted with the red box.

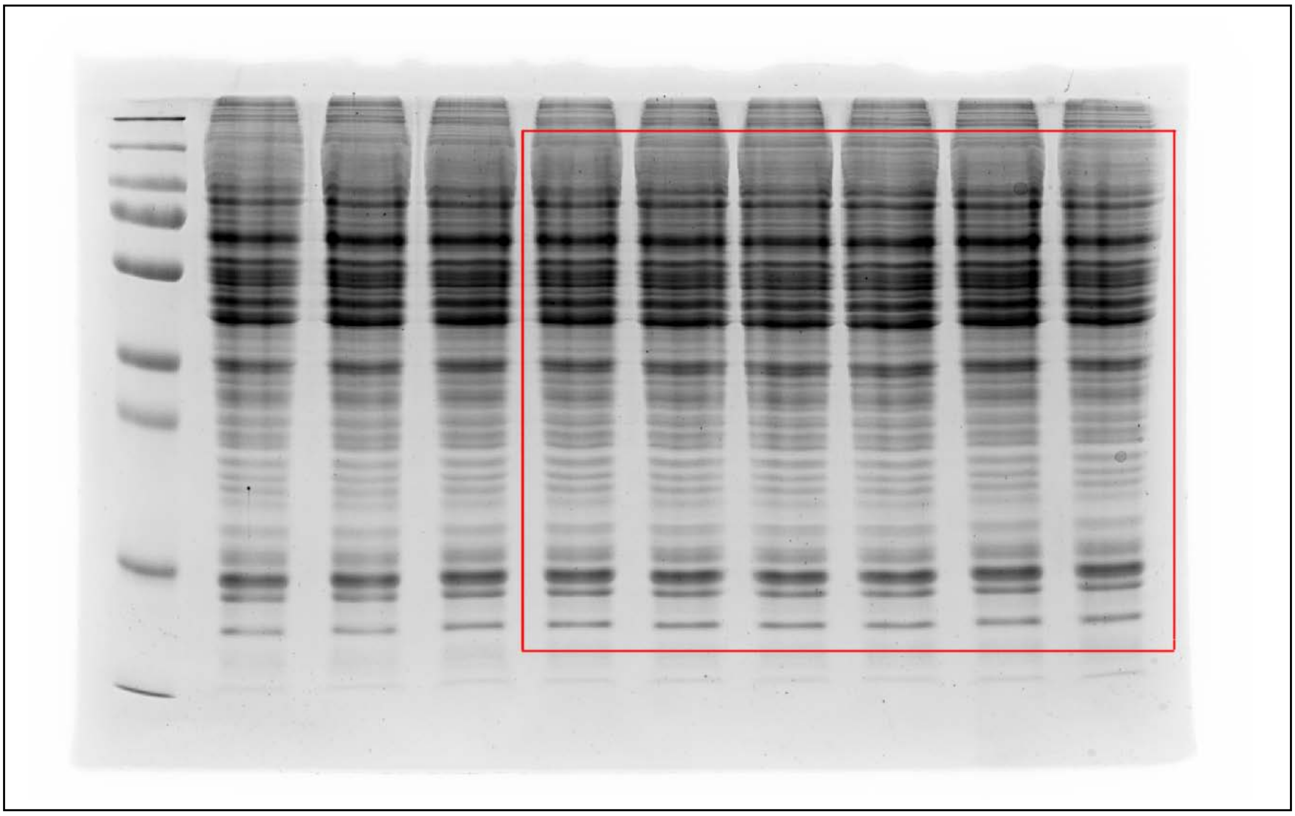

Supplementary Figure 10. Original image of the Figure 3B (radioautography) in the article. The region of the original image used in the main figure is denoted with the red box.

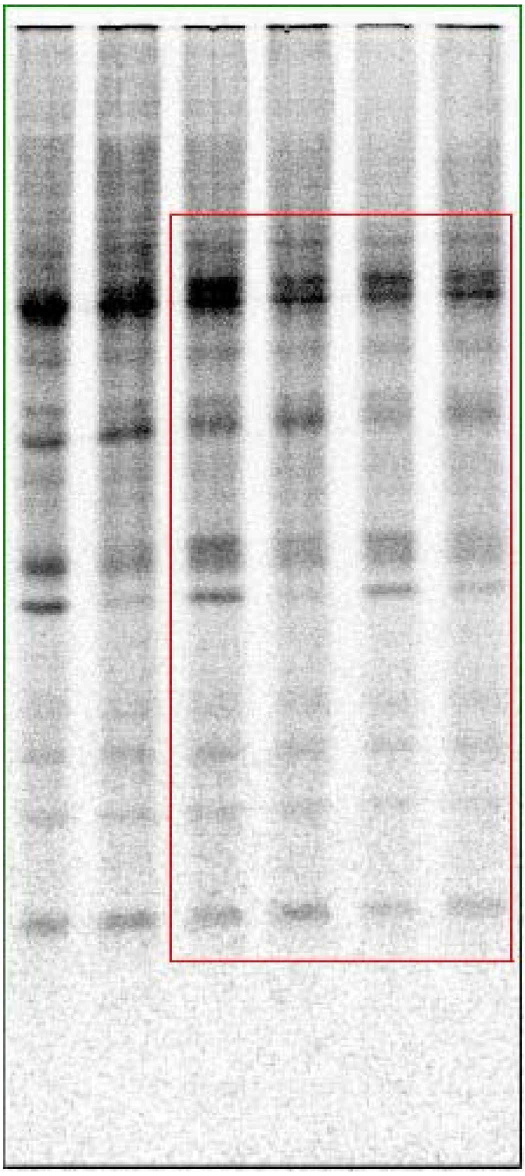

Supplementary Figure 11. Original image of the Figure 3B (Coomassie staining) in the article. The region of the original image used in the main figure is denoted with the red box.

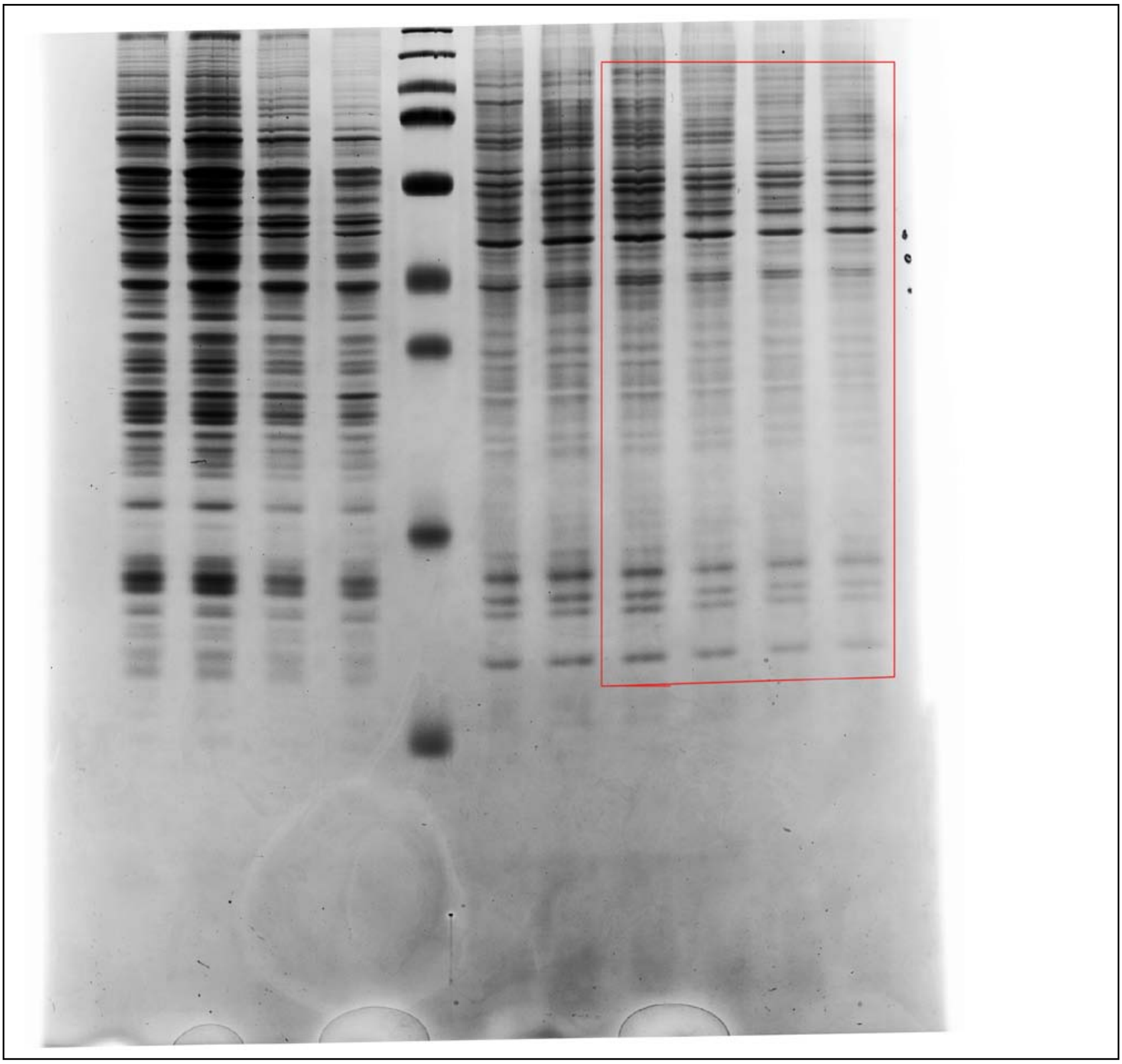

Supplement: Supplementary file 1 — Supplementary information. [file 41598_2020_64139_MOESM1_ESM.pdf]
